# Monitoramento da Temperatura Esofágica durante a Ablação de Fibrilação Atrial: Um Estudo Randomizado

**DOI:** 10.36660/abc.20250056

**Published:** 2025-07-10

**Authors:** Daniel Moreira Costa Moura, Renner Augusto Raposo Pereira, Cristiano Faria Pisani, Tan Chen Wu, Muhieddine Omar Chokr, Carina Abigail Hardy, Sissy Lara de Melo, Francisco Carlos da Costa Darrieux, Denise Tessariol Hachul, Mauricio Ibrahim Scanavacca

**Affiliations:** 1 Instituto do Coração do Hospital das Clínicas Faculdade de Medicina Universidade de São Paulo São Paulo SP Brasil Unidade Clínica de Arritmias, Instituto do Coração do Hospital das Clínicas da Faculdade de Medicina da Universidade de São Paulo, São Paulo, SP – Brasil

**Keywords:** Fibrilação Atrial, Fístula Esofágica, Ablação por Radiofrequência, Ensaio Clínico

## Abstract

**Fundamento:**

O isolamento das veias pulmonares (IVP) para a ablação da fibrilação atrial (FA) apresenta risco de lesão térmica esofágica (LTE), que pode levar a complicações graves.

**Objetivo:**

Avaliar três estratégias de monitoramento da temperatura esofágica luminal (TEL) e analisar sua eficácia na redução da incidência de LTE.

**Métodos:**

Pacientes com FA foram randomizados em três grupos de IVP, conforme a estratégia de monitoramento da temperatura: sem monitoramento de TEL (Grupo 1), monitoramento de TEL com termômetro unipolar (Grupo 2) e monitoramento de TEL com termômetro multipolar (Grupo 3). No Grupo 1, a ablação da FA foi realizada com potência fixa de 20 W na parede posterior do átrio esquerdo. Nos Grupos 2 e 3, a potência da ablação foi ajustada com base nas medições da TEL, utilizando como limite a temperatura de 37,5 °C. Cada grupo incluiu 20 pacientes. Considerou-se estatisticamente significativo um valor de p bilateral <0,05. O estudo foi registrado no ClinicalTrials.gov (#NCT03645070) e na International Clinical Trials Registry Platform (#RBR-2yvgyf).

**Resultados:**

Todos os pacientes foram submetidos a IVP e à esofagogastroduodenoscopia. Nenhum caso de LTE foi observado em pacientes monitorados com o termômetro multipolar. Em contraste, cinco pacientes sem monitoramento de TEL e seis pacientes monitorados com o termômetro unipolar desenvolveram LTE (p=0,006). Temperaturas mais elevadas foram registradas com o termômetro multipolar (37,9 vs 38,45 °C, p=0,018). Não houve diferenças significativas na duração do IVP ou no tempo total de aplicação da radiofrequência (p=0,250 e p=0,253, respectivamente).

**Conclusões:**

O monitoramento da TEL com termômetro multipolar durante o IVP reduz significativamente a incidência de LTE em comparação com a ausência de monitoramento ou o monitoramento com termômetro unipolar. A implementação de estratégias avançadas de monitoramento da TEL pode aumentar a segurança do paciente sem comprometer a eficiência do procedimento.

## Graphical abstract

**Figura Central:**
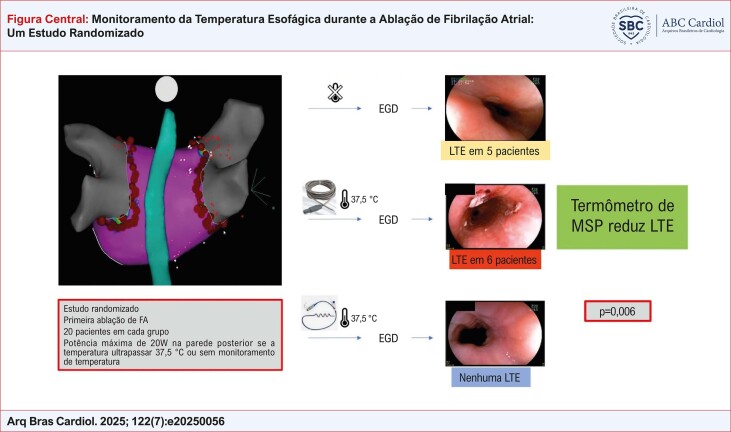
: Monitoramento da Temperatura Esofágica durante a Ablação de Fibrilação Atrial: Um Estudo Randomizado

AF: fibrilação atrial; EGD: esofagogastroduodenoscopia; LTE: lesão térmica esofágica; MSP: termômetro multipolar.

## Introdução

A ablação por cateter é uma estratégia bem estabelecida para o controle do ritmo em pacientes com fibrilação atrial (FA) e, atualmente, apresenta taxas de complicação aceitáveis.^1-3^ No entanto, a formação de uma fístula atrioesofágica, embora rara, está associada a alta taxa de mortalidade.^4-7^ Embora o mecanismo exato de formação da fístula atrioesofágica permaneça controverso, acredita-se que a lesão térmica direta desempenhe um papel importante. A lesão térmica esofágica (LTE), detectada por meio da esofagogastroduodenoscopia (EGD) após o isolamento das veias pulmonares (IVP), pode evoluir para perfuração esofágica e, consequentemente, para fístula atrioesofágica.^8,9^

Diversas estratégias têm sido empregadas para minimizar a lesão esofágica durante as aplicações de radiofrequência (RF). Dentre essas, apenas o monitoramento da temperatura esofágica luminal (TEL) e a redução da potência entregue à parede posterior são amplamente recomendados, embora essa ainda seja uma área de incerteza.^10-15^ Diversas sondas de TEL estão disponíveis comercialmente, variando em design, tamanho e resposta térmica transitória. A precisão do monitoramento de TEL na detecção do aquecimento da parede esofágica depende da distância entre a sonda de temperatura e o local da ablação.^16^ Resultados variáveis têm sido relatados quanto ao monitoramento de TEL e seu papel na redução da LTE.^17-20^ Embora o termômetro multipolar apresente maior sensibilidade na detecção do aumento de temperatura, ainda não foi claramente demonstrada a redução da incidência de LTE com o uso desse tipo de sonda.^21-23^

Portanto, o objetivo deste estudo foi avaliar a incidência de LTE após a ablação de FA utilizando três diferentes estratégias de prevenção da LTE.

## Materiais e métodos

### Desenho do estudo

Este foi um estudo piloto randomizado, paralelo, aberto e de centro único, que incluiu 60 pacientes com FA. Os pacientes foram alocados em três grupos na proporção de 1:1:1, utilizando uma lista randomizada baseada na web: Grupo 1, sem monitoramento de temperatura; Grupo 2, monitoramento com termômetro unipolar (Braile Biomédica, Brasil); e Grupo 3, monitoramento com termômetro multipolar (S-Cath™, CIRCA Scientific, LLC, Englewood, CO, EUA). O estudo foi aprovado pelo Comitê de Ética em Pesquisa de nossa instituição, e todos os procedimentos seguiram os princípios da Declaração de Helsinki. O estudo foi registrado na plataforma ClinicalTrials.gov do *National Institutes of Health* dos EUA (NCT03645070) e na *International Clinical Trials Registry Platform* (ICTRP: RBR-2yvgyf). Todos os participantes assinaram o termo de consentimento livre e esclarecido.

### Seleção de pacientes

Foram avaliados para elegibilidade todos os pacientes com idade entre 18 e 75 anos, com FA paroxística ou persistente, que se apresentaram em nossa instituição para ablação de FA utilizando o sistema de mapeamento eletroanatômico CARTO™ 3 (Biosense Webster, Inc., Diamond Bar, CA, EUA) entre julho de 2017 e outubro de 2018. Foram excluídos os pacientes que apresentavam trombo no átrio esquerdo (avaliado por ecocardiografia transesofágica), histórico de ablação prévia de FA ou cirurgia cardíaca com abertura do tórax, contraindicações ao uso de anticoagulantes, classe funcional III ou IV da *New York Heart Association*, acidente vascular cerebral nos últimos 3 meses, gravidez, histórico de distúrbios de coagulação, cirurgia esofágica prévia ou doença renal crônica avançada (creatinina >2,5 mg/dl).

### Procedimento de ablação de FA

Todos os pacientes incluídos foram submetidos a IVP antral utilizando o cateter Thermocool Smarttouch™ SF (Biosense Webster). Os procedimentos foram realizados sob anestesia geral. Nos Grupos 2 e 3, o termômetro foi posicionado antes do acesso venoso. O acesso transseptal duplo ao átrio esquerdo foi obtido utilizando a técnica padrão e introdutores padrão. Heparina intravenosa foi administrada para manter o tempo de coagulação ativado entre 250 e 350 segundos, monitorado a cada 30 minutos durante o procedimento. O IVP ipsilaterais foi realizado em bloco. Um cateter circular de mapeamento das veias pulmonares (Inquiry, Abbott) foi utilizado para verificar o IVP por meio dos testes de bloqueio de entrada e saída. Adenosina foi administrada para detectar condução dormente. Durante as aplicações na parede posterior, buscou-se manter uma força de contato de aproximadamente 10 g, e o Ablation Index (Biosense Webster) não estava disponível.

A potência de ablação foi limitada a um máximo de 30 W, utilizando a técnica de movimentação do cateter (*dragging*) e uma taxa máxima de irrigação de 17 ml/min. De acordo com o grupo do estudo, a energia máxima entregue na parede posterior variou. No Grupo 1, todas as ablações na parede posterior foram realizadas com 20 W, e o cateter foi mantido no mesmo ponto por no máximo 20 segundos. Nos Grupos 2 e 3, caso a TEL aumentasse para 37,5 °C, a ablação era interrompida, a potência máxima era reduzida para 20 W, e a ablação era retomada após a TEL cair abaixo de 37,0 °C. Nos locais em que a TEL aumentava, o cateter era deslocado para o ponto seguinte dentro de 20 segundos.

O termômetro unipolar tinha design linear, com um único termopar localizado na extremidade distal da sonda, e era eletricamente isolado. No Grupo 2, a posição da ponta do termômetro era ajustada sob orientação fluoroscópica para mantê-la o mais próximo possível da ponta do cateter de ablação. O termômetro multipolar possuía 12 sensores de temperatura distribuídos uniformemente ao longo da sonda, eletricamente isolados com elastômeros termoplásticos, e apresentava um corpo flexível com conformação em “S”. Quando posicionado, os sensores cobriam toda a parede posterior, eliminando a necessidade de ajustes posteriores.

### Avaliação pós-procedimento e seguimento

Todos os pacientes foram submetidos a EGD dentro de 3 dias após o procedimento, após pelo menos 6 horas de jejum, para avaliar a presença e a extensão da LTE. Um inibidor da bomba de prótons foi prescrito a todos os pacientes por quatro semanas. Os investigadores responsáveis pela EGD pós-ablação estavam cegos para os aspectos técnicos dos procedimentos de ablação. Todas as lesões na porção do esôfago adjacente ao átrio esquerdo e localizadas na parede anterior foram consideradas relacionadas à ablação e classificadas em cinco grupos: 0, ausência de lesão; 1, eritema; 2, hematoma; 3, erosão; e 4, úlcera. Caso fosse identificada LTE, uma nova EGD era realizada entre sete e dez dias após a avaliação inicial.

As avaliações de seguimento foram realizadas por telefone aos 30 dias e, posteriormente, a cada 6 meses. Durante essas ligações, era feita uma avaliação clínica geral e os pacientes eram agendados para a realização de eletrocardiograma e exame Holter 24 horas. A recorrência foi definida como qualquer episódio de FA ou taquicardia atrial com duração de 30 segundos ou mais, ocorrendo três meses após o procedimento de ablação.

### Desfechos

O desfecho primário foi a incidência de LTE avaliada por EGD. Os desfechos secundários incluíram a taxa de IVP, a duração da ablação de FA (medida como o tempo entre a primeira e a última lesão de ablação no átrio esquerdo), o tempo total de aplicação de RF e a TEL máxima alcançada durante o procedimento.

### Análise estatística

A normalidade dos dados foi avaliada utilizando o teste de Kolmogorov-Smirnov. Dados com distribuição não simétrica são apresentados como medianas e intervalos interquartílicos. Variáveis categóricas são apresentadas como frequências e porcentagens. Dependendo do tipo de dado analisado, a análise univariada foi realizada utilizando o teste da razão de verossimilhança, o teste de Kruskal-Wallis, o teste U de Wilcoxon-Mann-Whitney ou o teste χ^2^. A incidência de lesões esofágicas e de lesões esofágicas graves (graus 3 e 4) foi comparada utilizando o teste da razão de verossimilhança. Valores de p bilaterais <0,05 foram considerados estatisticamente significativos. Todas as análises estatísticas foram realizadas utilizando o software SPSS versão 18.0 para Windows.

## Resultados

### Características dos pacientes

**Figura 1:**
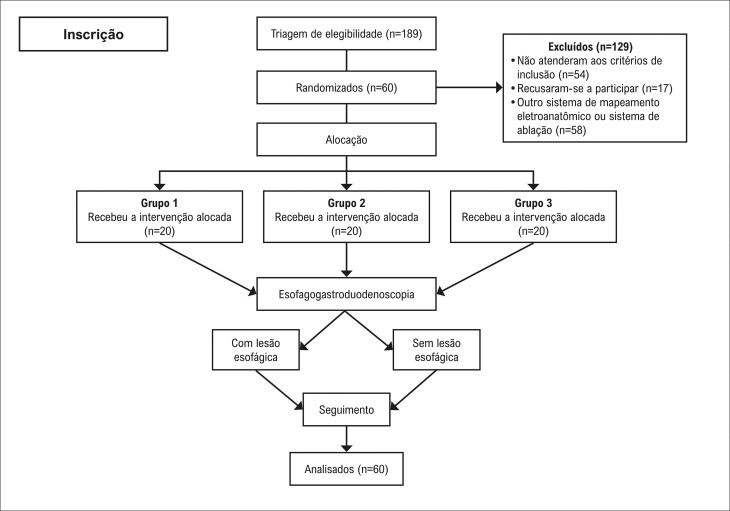
– Fluxograma do processo de seleção do estudo.

Dos 189 pacientes consecutivos avaliados para elegibilidade, 129 foram excluídos, e 60 foram randomizados para um dos três braços do estudo (Figura 1). Todos os pacientes foram submetidos a IVP e EGD. A maioria dos pacientes era do sexo masculino (95% vs 85% vs 65%, p=0,04), com idade mediana de 58 ou 59 anos (p=0,938), apresentava FA paroxística (p=0,053) e tinha diâmetro atrial esquerdo variando de 41 a 43 mm (p=0,815). Não foram observadas diferenças significativas entre os grupos quanto à prevalência de hipertensão, diabetes, histórico de insuficiência cardíaca ou fração de ejeção do ventrículo esquerdo. As características demográficas dos pacientes estão resumidas na Tabela 1.

**Tabela 1:** – Características basais

	Grupo 1 (n=20)	Grupo 2 (n=20)	Grupo 3 (n=20)	Valor p
Sexo masculino, n (%)	19 (95)	17 (85)	13 (65)	0,040
Idade, anos (Q1; Q3)	59 (52; 63)	58 (46; 64)	58 (51; 65)	0,938
IMC, kg/m^2^ (Q1; Q3)	27,6 (25,9; 29,9)	28,6 (26,7; 31,3)	29,5 (26,5; 33,1)	0,413
Classificação da FA				0,530
FA paroxística, n (%)	15 (75)	18 (90)	16 (80)	
FA persistente, <1 ano, n (%)	3 (15)	0 (0)	4 (20)	
FA persistente, >1 ano, n (%)	2 (10)	2 (10)	0 (0)	
Hipertensão, n (%)	8 (40)	6 (30)	8 (40)	0,750
Diabetes, n (%)	2 (10)	1 (5)	2 (10)	0,789
Insuficiência cardíaca, n (%)	0 (0)	2 (10)	1 (5)	0,237
Escore CHA_2_DS_2_-VASc >2, n (%)	4 (20)	4 (20)	9 (45)	0,350
Cardioversão elétrica prévia, n (%)	6 (30)	3 (15)	7 (35)	0,330
Anticoagulação, n (%)	13 (65)	13 (65)	16 (80)	0,490
Uso de antiarrítmicos, n (%)	16 (80)	14 (70)	13 (65)	0,563
Uso prévio de antiarrítmicos, n (%)	10 (50)	6 (30)	8 (40)	0,435
Diâmetro do átrio esquerdo, mm (Q1; Q3)	43 (36; 47)	42 (38; 45)	41 (39; 48)	0,815
Fração de ejeção do ventrículo esquerdo, % (Q1; Q3)	64 (60; 66)	64 (59; 68)	65 (62; 67)	0,650

FA: fibrilação atrial; IMC: índice de massa corporal.

### Procedimento de ablação e monitoramento da TEL

O IVP foi alcançado em todos os pacientes por meio de ablação no nível do antro, exceto em um paciente do Grupo 3 que apresentava extensa área de fibrose na parede posterior do átrio esquerdo; nesse caso, o IVP foi concluído com o isolamento em bloco da parede posterior. A critério do operador, lesões adicionais na carina direita e/ou esquerda foram realizadas em pacientes de todos os grupos, sem diferenças significativas entre os grupos (carina direita, p=0,333; carina esquerda, p=0,839).

A TEL basal foi semelhante entre os dois grupos com monitoramento de TEL. Um aumento da TEL foi observado em ambos os grupos, com temperaturas máximas mais elevadas registradas com o termômetro MSP (37,9 vs 38,45 °C, p=0,018). Entre os pacientes com monitoramento de TEL, o aumento de temperatura ocorreu em ambos os lados em 35% dos casos. Em três pacientes do Grupo 3 e quatro pacientes do Grupo 2, a potência da RF foi reduzida para 10 W durante a ablação no local de maior elevação da TEL devido ao aumento rápido da temperatura (p=0,677).

Embora o aumento da TEL tenha ocorrido com mais frequência no Grupo 3 do que no Grupo 2, e não houvesse monitoramento de TEL no Grupo 1, não foram encontradas diferenças significativas no tempo para alcançar o IVP ou no tempo total de aplicação de RF (p=0,250 e p=0,253, respectivamente). As características do procedimento estão resumidas na Tabela 2.

**Tabela 2:** – Dados do procedimento

	Grupo 1 (n=20)	Grupo 2 (n=20)	Grupo 3 (n=20)	Valor p
IVP, n (%)	20 (100)	20 (100%)	20 (100%)	
Aplicações de RF na carina direita, n (%)	5 (25)	2 (10)	5 (26,3)	0,333
Aplicações de RF na carina esquerda, n (%)	7 (35)	6 (30)	5 (26,3)	0,839
Bloqueio do istmo cavo-tricúspide, n (%)	8 (40)	6 (30)	10 (52,6)	0,355
Tempo até o IVP, min (Q1; Q3)	78 (57; 105)	68 (61; 91)	63 (55; 80)	0,250
Tempo de RF, min (Q1; Q3)	31,8 (26,9; 36,5)	37,5 (28,6; 41,8)	34,7 (28,8; 42,3)	0,253
TEL inicial, °C (Q1; Q3)		36,2 (35,9; 36,4)	36,3 (35,6; 36,5)	0,848
TEL máxima, °C (Q1; Q3)		37,9 (37,5; 38,8)	38,4 (38; 39,4)	0,018
Local de aumento da TEL, n (%)				0,290
Veias pulmonares direitas, n (%)		9 (45)	5 (25)	
Veias pulmonares esquerdas, n (%)		4 (20)	8 (40)	
Ambas as veias pulmonares, n (%)		7 (35)	7 (35)	
Aplicação de 10 W, n (%)		4 (20)	3 (15)	0,677

IVP: isolamento das veias pulmonares; RF: radiofrequência; TEL: temperatura esofágica luminal.

Apenas um evento adverso grave foi registrado: tamponamento cardíaco, que exigiu drenagem percutânea, com boa recuperação do paciente.

### Procedimento de EGD

Todos os pacientes foram submetidos à EGD. Os principais achados, ilustrados na Figura Central, demonstram que o uso do termômetro multipolar no Grupo 3 durante os procedimentos de IVP resultou na ausência de casos de LTE (p=0,006). Mesmo ao comparar os três grupos considerando apenas as lesões mais graves (graus 3 e 4), a diferença permaneceu estatisticamente significativa em favor do Grupo 3 (p=0,029).

**Figura 2:**
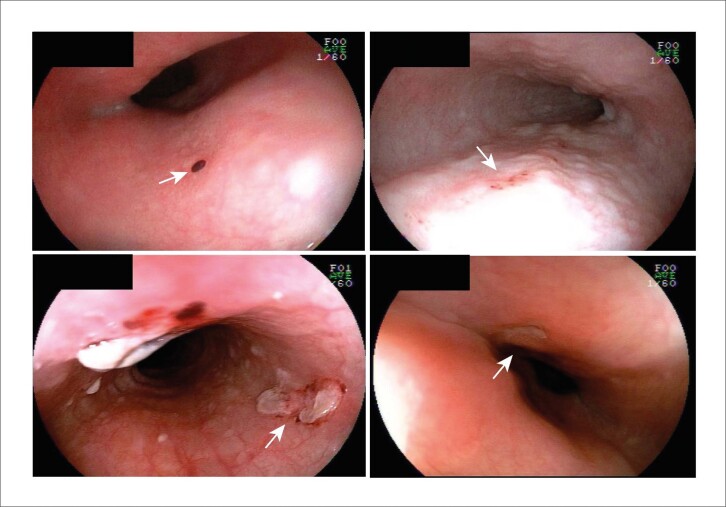
– A partir do canto superior esquerdo, no sentido horário: lesão térmica esofágica, grau II; lesão térmica esofágica, grau II; lesão térmica esofágica, grau IV; lesão térmica esofágica, grau III.

No Grupo 1, LTE foi identificada em cinco pacientes (25%): uma lesão de grau II, duas lesões de grau III e duas lesões de grau IV. As lesões de grau III mediam, respectivamente, 4 mm e 2,5 mm de diâmetro, e as de grau IV, 6 mm e 1 mm. No Grupo 2, um paciente apresentou duas lesões de LTE de grau III, medindo 10 mm e 12 mm de diâmetro, respectivamente, e outros cinco pacientes apresentaram lesões de LTE: dois com lesões de grau II, dois com lesões de grau III medindo, respectivamente, 4 mm e 5 mm, e um com lesão de grau IV medindo 7 mm. A Figura 2 apresenta exemplos dos achados de LTE.

Alimentos foram encontrados no estômago, apesar do jejum, em pelo menos 25% dos pacientes, e esofagite foi identificada em 10% dos pacientes, sem diferenças significativas entre os grupos (p=0,525 e p=0,098, respectivamente). Nenhuma outra lesão foi detectada. Uma nova EGD foi realizada em todos os pacientes com LTE sete dias após o exame inicial, e, em todos os casos, as lesões haviam se resolvido. Os achados da EGD estão resumidos na Tabela 3.

**Tabela 3:** – Resultados da esofagogastroduodenoscopia

	Grupo 1 (n=20)	Grupo 2 (n=20)	Grupo 3 (n=20)	Valor p
LTE, n (%)	5 (25)	6 (30)	0 (0)	0,006
Grau I	0 (0)	0 (0)	0 (0)	
Grau II	1 (5)	2 (10)	0 (0)	0,250
Grau III	2 (10)	3 (15)	0 (0)	0,115
Grau IV	2 (10)	1 (5)	0 (0)	0,250
Graus III e IV, n (%)	4 (20)	4 (20)	0 (0)	0,029
Presença de alimento no estômago, n (%)	5 (25)	7 (35)	8 (40)	0,525
Esofagite, n (%)	2 (10)	2 (10)	2 (10)	0,998

LTE: lesão térmica esofágica.

### Seguimento

Exceto por dois pacientes do Grupo 2 e dois do Grupo 3, todos os pacientes foram acompanhados por uma mediana de 27,8 meses (Q1:Q3, 24,6:31,4). Um total de 13 pacientes (22%) apresentou recorrência de FA: três pacientes no Grupo 1, seis no Grupo 2 e quatro no Grupo 3 (p=0,408). Não foram encontradas diferenças significativas na recorrência de FA em relação ao tempo para alcançar o IVP (p=0,648), ao tempo total de aplicação de RF (p=0,480), à classificação da FA (p=0,451), ao tamanho do átrio esquerdo (p=0,928), à potência mínima da RF (p=0,658), à temperatura local máxima (p=0,300) ou à TEL máxima (p=0,637).

## Discussão

Até onde sabemos, este é o primeiro estudo randomizado que compara as três estratégias de monitoramento de TEL mais utilizadas para a redução da LTE: ausência de monitoramento de TEL com redução da potência de ablação, monitoramento com termômetro unipolar e monitoramento com termômentro multipolar. Um limite de temperatura baixo (37,5 °C) foi utilizado para a interrupção das aplicações de RF, e todos os pacientes foram submetidos a EGD.

### TEL vs ausência de TEL

Diversos estudos relataram uma ampla variação na incidência de LTE durante a ablação de FA ao comparar o monitoramento de TEL com a ausência de monitoramento. No entanto, esses estudos mostraram resultados divergentes e nenhum deles foi randomizado.^9,18-20,24-28^

Kiuchi et al. e Müller et al.^18,20^ utilizaram um termômetro multipolar (SensiTherm™) e relataram resultados conflitantes: o estudo de Müller encontrou uma maior incidência de LTE no grupo com monitoramento de temperatura (30% vs 2,5%), enquanto o estudo de Kiuchi relatou uma incidência menor (0% vs 7,5%). Mais recentemente, Schoene et al.,^29^ no ensaio clínico randomizado OPERA, investigaram se o uso de um termômetro multipolar (SensiTherm™) era não inferior à ablação de FA sem monitoramento de TEL. Eles encontraram uma incidência de LTE semelhante entre os grupos (11,1% vs 8,9%). No estudo, foi utilizado um limite de temperatura bastante elevado (41 °C) para interromper as aplicações de RF, além de ajustes de potência diferentes (25 a 30 W vs 25 W), o que resultou em uma diferença significativa na energia de RF entregue à parede posterior (p<0,001).

Meininghaus et al.^30^ conduziram um ensaio randomizado comparando IVP com monitoramento de TEL utilizando a sonda CIRCA S-CATH™ versus ausência de monitoramento. Todos os pacientes foram submetidos à EGD antes e depois do IVP, que foi realizado com potência de 25 W e estratégia de redução de potência guiada pela TEL utilizando como limite a temperatura de 41 °C. Neste estudo, lesões de mucosa foram observadas em seis de 44 pacientes no grupo com monitoramento de TEL e em dois de 42 pacientes no grupo controle. Entretanto, o valor de p não foi significativo.

### Tipo de sonda

Turagam et al.^16^ avaliaram as características termodinâmicas de 22 sondas de TEL disponíveis comercialmente e demonstraram uma variação notavelmente ampla entre elas. A sonda CIRCA S-CATH™ apresentou um perfil termodinâmico superior em comparação às demais. Em um estudo prospectivo não randomizado, incluindo 20 pacientes com FA sintomática submetidos ao IVP inicial, Tschabrunn et al.^23^ mostraram que a CIRCA S-CATH™ teve desempenho termodinâmico superior em comparação a uma sonda unipolar linear padrão e não defletível.

Em um estudo prospectivo não randomizado, Carroll et al.^21^ investigaram as taxas de LTE associadas ao uso de uma sonda multipolar (CIRCA S-CATH™) versus uma sonda unipolar (Acoustascope, Smiths Medical ASD, Inc., Keene, NH, EUA). Foi aplicada uma potência de 25 W na parede posterior, com um limite de temperatura de 38 °C. Entre os 543 pacientes incluídos (455 com unipolar e 88 com multipolar), apenas aqueles que apresentaram TEL máxima superior a 39 °C foram submetidos à endoscopia (75% dos pacientes no grupo multipolar e 39% no grupo unipolar). Uma taxa significativamente maior de ulceração esofágica foi associada ao uso do termômetro multipolar (46% vs 29%, p=0,02). No entanto, observou-se uma diferença significativa nas características dos pacientes, com o grupo multipolar apresentando maior tempo total de ablação por RF e maior energia total aplicada.

Em um estudo randomizado envolvendo cem pacientes, Kuwahara et al.^22^ compararam um termômetro unipolar defletível com um termômetro multipolar (SensiTherm™). Foi utilizado um cateter de ponta irrigada aberta sem sensor de força de contato, com configuração de RF de 25 a 30 W e limite de temperatura de 45 °C, aplicando um corte de 42 °C. Não foi encontrada diferença significativa nas taxas de LTE entre os grupos (30% vs 20%, p=0,25).

### Nível de energia

O nível de energia aplicado na parede posterior em nosso estudo reflete a estratégia padrão de ablação utilizada nos últimos anos.^29,31,32^ No entanto, a ablação de alta potência e curta duração ganhou popularidade recentemente. Chieng et al.^33^ publicaram o estudo Hi-Lo HEAT, no qual não encontraram diferença nas taxas de LTE entre as estratégias de ablação com 40 W e 25 W. Chen et al.^24^ estudaram 120 pacientes consecutivos, comparando o monitoramento de TEL com a sonda CIRCA S-CATH™ versus ausência de monitoramento de TEL, utilizando um limite de 39 °C para interrupção das aplicações de RF. A potência da RF foi ajustada para 50 W durante todo o procedimento, com taxa de irrigação de 20 ml/min, seguindo um modelo de controle de potência e guiado pelo *ablation index* (Biosense Webster), com valor alvo de 400 na parede posterior do átrio esquerdo. Eles relataram baixa incidência de LTE, sem diferença significativa entre os grupos (3,3% vs 1,7%, p=0,99).

### Limitações

Nosso estudo apresenta várias limitações. Foi um estudo piloto, de centro único, com pequeno tamanho amostral. A maioria dos pacientes apresentava FA paroxística, escore CHA_2_DS_2_-VASc <2 e ausência de dilatação significativa do átrio esquerdo. Não se pode excluir a possibilidade de que o dano esofágico tenha sido causado pela inserção e manipulação da sonda de temperatura nos Grupos 2 e 3, ou pela sonda de ecocardiografia transesofágica. Como nenhuma EGD foi realizada antes da ablação por RF, podemos apenas presumir que a localização das lesões na parede anterior do esôfago, distante da junção gastroesofágica, torna provável que a LTE esteja relacionada à ablação de FA.

Não correlacionamos especificamente os achados locais de LTE com os dados de ablação por RF, informações de força de contato do sistema de mapeamento eletroanatômico ou medições de temperatura. Apenas um sistema de mapeamento eletroanatômico foi utilizado e diferentes resultados poderiam ser observados com outros sistemas. Todos os procedimentos de ablação foram realizados sob anestesia geral. Em relação à técnica de ablação, nem o Visitag (Biosense Webster) nem a estratégia de ablação de alta potência e curta duração estavam disponíveis à época, e o isolamento da parede posterior foi realizado em apenas um paciente. Utilizamos uma técnica de movimentação contínua (*dragging*) do cateter com 30 W, e, embora essa abordagem tenha sido empregada, tanto a taxa de recorrência quanto a incidência de lesões esofágicas permaneceram baixas, o que poderia ter sido diferente caso estratégias alternativas fossem usadas.

Com relação à recorrência da FA, quatro pacientes foram perdidos no seguimento — dois no Grupo 2 e dois no Grupo 3 —, e a recorrência de FA foi avaliada apenas por meio de eletrocardiograma e exame Holter 24 horas.

Não há consenso quanto ao limite ideal de temperatura para a prevenção de LTE, e diferentes limites de temperatura não foram comparados neste estudo.

## Conclusão

Este estudo piloto demonstra que o monitoramento da TEL com o uso de termômetro multipolar reduz significativamente a incidência de LTE em comparação ao monitoramento com termômetro unipolar ou à ausência de monitoramento, sem aumento da taxa de recorrência de FA após a ablação por RF. Esses achados sugerem que o uso do termômetro multipolar pode aumentar a segurança do paciente durante o IVP, minimizando efetivamente o risco de LTE. No entanto, são necessários novos estudos randomizados, com amostras maiores, para confirmar esses resultados e orientar a prática clínica futura.
