# Supplementary material for: Monitoramento da Temperatura Esofágica durante a Ablação de Fibrilação Atrial: Um Estudo Randomizado
Source: Arq Bras Cardiol. 2025 Jul 10;122(7):e20250056. [Article in Portuguese] doi: 10.36660/abc.20250056 (PMC12266716; doi:10.36660/abc.20250056)
Supplement: Translation

# Translation: Esophageal Temperature Monitoring during Atrial Fibrillation Ablation: A Randomized Study

**DOI:** 10.36660/abc.20250056i

**Authors:** Daniel Moreira Costa Moura, Renner Augusto Raposo Pereira, Cristiano Faria Pisani, Tan Chen Wu, Muhieddine Omar Chokr, Carina Abigail Hardy, Sissy Lara de Melo, Francisco Carlos da Costa Darrieux, Denise Tessariol Hachul, Mauricio Ibrahim Scanavacca

**Affiliations:** 1 Brazil Unidade Clínica de Arritmias, Instituto do Coração do Hospital das Clínicas da Faculdade de Medicina da Universidade de São Paulo, São Paulo, SP – Brazil

**Keywords:** Atrial Fibrillation, Esophageal Fistula, Radiofrequency Ablation, Clinical Trial

## Abstract

**Background:**

Pulmonary vein isolation (PVI) for atrial fibrillation (AF) ablation carries a risk of esophageal thermal injury (ETI), which can lead to severe complications.

**Objective:**

To evaluate three luminal esophageal temperature (LET) monitoring strategies and assess their effectiveness in reducing the incidence of ETI.

**Methods:**

Patients with AF were randomized into three PVI groups according to the temperature monitoring strategy: no LET monitoring (Group 1), LET monitoring with a single-sensor probe (SSP) thermometer (Group 2), and LET monitoring with a multisensor probe (MSP) thermometer (Group 3). In Group 1, AF ablation was performed at a fixed power of 20 W on the left atrial posterior wall. In Groups 2 and 3, AF ablation power was titrated based on LET measurements, with a cutoff temperature of 37.5 °C. Each group included 20 patients. A two-sided p-value <0.05 was considered statistically significant. The trial was registered on ClinicalTrials.gov (#NCT03645070) and International Clinical Trials Registry Platform (#RBR-2yvgyf).

**Results:**

All patients underwent PVI and esophagogastroduodenoscopy. No ETI was observed in patients monitored with an MSP thermometer. In contrast, five patients without LET monitoring and six patients monitored with an SSP thermometer developed ETI (p=0.006). Higher temperatures were recorded with an MSP thermometer (37.9 vs. 38.45 °C, p=0.018). There were no significant differences in PVI duration or total radiofrequency application time (p=0.250 and p=0.253, respectively).

**Conclusions:**

LET monitoring with an MSP thermometer during PVI significantly reduces the incidence of ETI compared to no monitoring or SSP monitoring. Implementing advanced LET monitoring strategies may enhance patient safety without compromising procedural efficiency.

## Introduction

Catheter ablation is a well-established strategy for rhythm control in patients with atrial fibrillation (AF) and is currently associated with acceptable complication rates.^1-3^ However, the formation of an atrioesophageal fistula, although rare, carries a high mortality rate.^4-7^ Although the exact mechanism of atrioesophageal fistula formation remains controversial, direct thermal injury appears to play a major role. Esophageal thermal injury (ETI) detected via esophagogastroduodenoscopy (EGD) following pulmonary vein isolation (PVI) has been shown to progress to esophageal perforation and, ultimately, atrioesophageal fistula.^8,9^

Many strategies have been employed to minimize esophageal injury during radiofrequency (RF) applications. Of these, only luminal esophageal temperature (LET) monitoring and reduced power delivery to the posterior wall are widely recommended, although this remains an area of uncertainty.^10-15^ Several LET probes are commercially available, differing in design, size, and transient thermal response. The accuracy of LET monitoring in detecting esophageal wall heating depends on the distance between the temperature probe and the ablation site.^16^ Variable results regarding LET monitoring and its role in reducing ETI have been reported.^17-20^ Although a multisensor probe (MSP) thermometer demonstrates greater sensitivity in detecting temperature increases, a reduction in ETI incidence with the use of this type of probe has not yet been clearly demonstrated.^21-23^

Therefore, the aim of this study was to evaluate the incidence of ETI following AF ablation using three different strategies for ETI prevention.

## Materials and methods

### Study design

This was a single-center, open-label, randomized, parallel pilot trial that included 60 patients with AF. Patients were allocated to three groups in a 1:1:1 ratio using a randomized web-based list: Group 1, no temperature monitoring; Group 2, SSP thermometer (Braile Biomédica, Brazil) monitoring; and Group 3, MSP thermometer (S-Cath™, CIRCA Scientific, LLC, Englewood, CO, USA) monitoring. The study was approved by the Research Ethics Board of our institution, and all procedures adhered to the tenets of the Declaration of Helsinki. The trial was registered on the U.S. National Institutes of Health (ClinicalTrials.gov: NCT03645070) and the International Clinical Trials Registry Platform (ICTRP: RBR-2yvgyf). Written informed consent was obtained from all participants.

### Patient selection

We assessed the eligibility of all patients aged between 18 and 75 years with paroxysmal or persistent AF who presented to our institution for AF ablation using the CARTO™ 3 (Biosense Webster, Inc., Diamond Bar, CA, USA) electroanatomic mapping system between July 2017 and October 2018. Patients were excluded if they had thrombus in the left atrium (assessed by transesophageal echocardiography), prior AF ablation or open-chest cardiac surgery, contraindications to anticoagulation, New York Heart Association functional class III or IV, stroke within the past 3 months, pregnancy, a history of blood clotting abnormalities, prior esophageal surgery, or advanced chronic kidney disease (creatinine >2.5 mg/dL).

### AF ablation procedure

All included patients underwent wide antral PVI using a Thermocool Smarttouch™ SF Catheter (Biosense Webster). Procedures were performed under general anesthesia. In Groups 2 and 3, the thermometer was positioned before venous access. Double transseptal access to the left atrium was obtained using the standard technique and standard sheaths. Intravenous heparin was administered to maintain activated clotting times between 250 and 350 seconds, monitored every 30 minutes during the procedure. Isolation of the ipsilateral pulmonary veins was performed en bloc. A circular diagnostic pulmonary vein mapping catheter (Inquiry, Abbott) was used to verify PVI through entrance and exit block tests. Adenosine was administered to detect dormant conduction. During posterior wall applications, we aimed to maintain a contact force of approximately 10 g, and the Ablation Index (Biosense Webster) was not available.

Ablation power was limited to a maximum of 30 W, using a catheter dragging approach and a maximum irrigation rate of 17 mL/min. According to the study group, the maximum energy delivery at the posterior wall varied. In Group 1, all posterior wall ablations were performed with 20 W, and the catheter was maintained at the same point for no more than 20 seconds. In Groups 2 and 3, if LET rose to 37.5 °C, ablation was halted, the maximum energy delivery was reduced to 20 W, and ablation was resumed once LET dropped below 37.0 °C. At sites where LET increased, the catheter was moved to the next point within 20 seconds.

The SSP thermometer had a linear design with a single thermocouple located at the distal end of the probe, and it was electrically isolated. In Group 2, the position of the thermometer tip was adjusted under fluoroscopic guidance to keep it as close as possible to the tip of the ablation catheter. The MSP thermometer had 12 temperature sensors evenly spaced along the probe, electrically insulated with thermoplastic elastomers, and featured a smooth shaft with an “S” conformation. When positioned, the sensors covered the entire posterior wall, eliminating the need for further adjustment.

### Post-procedural evaluation and follow-up

All patients underwent EGD within 3 days after the procedure, following at least 6 hours of fasting, to assess the presence and extent of ETI. A proton pump inhibitor was prescribed to all patients for four weeks. Investigators performing the post-ablation EGD were blinded to the technical aspects of the ablation procedures. All lesions in the portion of the esophagus adjacent to the left atrium and located on the anterior wall were assumed to be ablation-related and were classified into five groups: 0, no lesion; 1, erythema; 2, hematoma; 3, erosion; and 4, ulcer. If ETI was identified, a repeat EGD was performed seven to ten days after the initial evaluation.

Follow-up assessments were conducted via telephone at 30 days and every 6 months thereafter. During these calls, a general clinical assessment was performed, and patients were scheduled for electrocardiogram and 24-hour Holter monitoring. Recurrence was defined as any episode of AF or atrial tachycardia lasting 30 seconds or longer, occurring three months after the ablation procedure.

### Outcomes

The primary outcome was the incidence of ETI as assessed by EGD. Secondary outcomes included the PVI rate, the duration of AF ablation (measured as the time between the first and last ablation lesion in the left atrium), total RF application time, and the maximum LET reached during the procedure.

### Statistical analysis

The normality of data was assessed using the Kolmogorov-Smirnov test. Data with a nonsymmetric distribution are reported as medians and interquartile ranges. Categorical variables are presented as frequencies and percentages. Depending on the type of data analyzed, univariate analysis was performed using the likelihood ratio test, Kruskal-Wallis test, Wilcoxon-Mann-Whitney U test, or χ^2^ test. The incidence of esophageal lesions and severe esophageal lesions (grades 3 and 4) was compared using the likelihood ratio test. Two-tailed p-values <0.05 were considered statistically significant. All statistical analyses were performed using SPSS version 18.0 for Windows.

## Results

### Patient characteristics

Of the 189 consecutive patients assessed for eligibility, 129 were excluded, and 60 were randomized into one of the three study arms (Figure 1). All patients underwent PVI and EGD. Most patients were male (95% vs. 85% vs. 65%, p=0.04), with a median age of 58 or 59 years (p=0.938), had paroxysmal AF (p=0.053), and had a left atrial diameter ranging from 41 to 43 mm (p=0.815). No significant differences were observed between groups regarding the prevalence of hypertension, diabetes, history of heart failure, or left ventricular ejection fraction. Patient demographic characteristics are summarized in Table 1.

**Figure 1:** – Flowchart of the study selection process.

**Table 1:** – Baseline characteristics

	Group 1 (n=20)	Group 2 (n=20)	Group 3 (n=20)	p-value
Male sex, n (%)	19 (95)	17 (85)	13 (65)	0.040
Age, years (Q1; Q3)	59 (52; 63)	58 (46; 64)	58 (51; 65)	0.938
BMI, kg/m^2^ (Q1; Q3)	27.6 (25.9; 29.9)	28.6 (26.7; 31.3)	29.5 (26.5; 33.1)	0.413
AF classification				0.530
Paroxysmal AF, n (%)	15 (75)	18 (90)	16 (80)	
Persistent AF, <1 year, n (%)	3 (15)	0 (0)	4 (20)	
Persistent AF, >1 years, n (%)	2 (10)	2 (10)	0 (0)	
Hypertension, n (%)	8 (40)	6 (30)	8 (40)	0.750
Diabetes, n (%)	2 (10)	1 (5)	2 (10)	0.789
Heart failure, n (%)	0 (0)	2 (10)	1 (5)	0.237
CHA2DS2-VASc score >2, n (%)	4 (20)	4 (20)	9 (45)	0.350
Previous electrical cardioversion, n (%)	6 (30)	3 (15)	7 (35)	0.330
Anticoagulation, n (%)	13 (65)	13 (65)	16 (80)	0.490
Use of antiarrhythmics, n (%)	16 (80)	14 (70)	13 (65)	0.563
Previous use of antiarrhythmics, n (%)	10 (50)	6 (30)	8 (40)	0.435
Left atrium diameter, mm (Q1; Q3)	43 (36; 47)	42 (38; 45)	41 (39; 48)	0.815
Left ventricular ejection fraction, % (Q1; Q3)	64 (60; 66)	64 (59; 68)	65 (62; 67)	0.650

AF: atrial fibrillation; BMI: body mass index.

### Ablation procedure and LET monitoring

PVI was achieved in all patients through antral ablation, except for one patient in Group 3 who had an extensive scar at the posterior wall of the left atrium; in this case, PVI was completed with box isolation of the posterior wall. At the operator’s discretion, additional lesions at the right and/or left carina were performed in patients from all groups, with no significant differences observed between groups (right carina, p=0.333; left carina, p=0.839).

The baseline LET was similar between both groups with LET monitoring. A rise in LET was observed in both groups, with higher maximum temperatures recorded with an MSP thermometer (37.9 vs. 38.45 °C, p=0.018). Among patients with LET monitoring, a temperature rise occurred on both sides in 35% of patients. In three patients from Group 3 and four patients from Group 2, RF power was reduced to 10 W during ablation at the site of the highest LET rise due to rapid temperature increase (p=0.677).

Although LET increased more frequently in Group 3 than in Group 2, and there was no LET monitoring in Group 1, no significant differences were found in the time to achieve PVI or in the total RF application time (p=0.250 and p=0.253, respectively). Procedural characteristics are summarized in Table 2.

**Table 2:** – Procedure data

	Group 1 (n=20)	Group 2 (n=20)	Group 3 (n=20)	p-value
PVI, n (%)	20 (100)	20 (100%)	20 (100%)	
RF applications at right carina, n (%)	5 (25)	2 (10)	5 (26.3)	0.333
RF applications at left carina, n (%)	7 (35)	6 (30)	5 (26.3)	0.839
Cavotricuspid isthmus block, n (%)	8 (40)	6 (30)	10 (52.6)	0.355
Time to PVI, min (Q1; Q3)	78 (57; 105)	68 (61; 91)	63 (55; 80)	0.250
RF time, min (Q1; Q3)	31.8 (26.9; 36.5)	37.5 (28.6; 41.8)	34.7 (28.8; 42.3)	0.253
Initial LET, °C (Q1; Q3)		36.2 (35.9; 36.4)	36.3 (35.6; 36.5)	0.848
Maximum LET, °C (Q1; Q3)		37.9 (37.5; 38.8)	38.4 (38; 39.4)	0.018
Site of LET rise, n (%)				0.290
Right pulmonary veins, n (%)		9 (45)	5 (25)	
Left pulmonary veins, n (%)		4 (20)	8 (40)	
Both pulmonary veins, n (%)		7 (35)	7 (35)	
10 W application, n (%)		4 (20)	3 (15)	0.677

LET: luminal esophageal temperature; PVI: pulmonary vein isolation; RF: radiofrequency.

Only one serious adverse event occurred: cardiac tamponade requiring percutaneous drainage, with the patient experiencing a good recovery.

### EGD procedure

All patients underwent EGD. The main findings, illustrated in the Central Illustration, demonstrate that the use of an MSP thermometer in Group 3 during PVI procedures resulted in no cases of ETI (p=0.006). Even when comparing the three groups regarding more severe lesions (grades 3 and 4), the difference remained statistically significant in favor of Group 3 (p=0.029).

In Group 1, ETI was identified in five patients (25%): one grade II lesion, two grade III lesions, and two grade IV lesions. The grade III lesions measured 4 mm and 2.5 mm in diameter, respectively, and the grade IV lesions measured 6 mm and 1 mm, respectively. In Group 2, one patient had two grade III ETI lesions measuring 10 mm and 12 mm in diameter, respectively, and five additional patients had ETI lesions: two with grade II lesions, two with grade III lesions measuring 4 mm and 5 mm, respectively, and one with a grade IV lesion measuring 7 mm. Figure 2 shows examples of ETI findings.

**Figure 2:** – From the top left, clockwise: esophageal thermal injury, grade II; esophageal thermal injury, grade II; esophageal thermal injury, grade IV; esophageal thermal injury, grade III.

Food was found in the stomach despite the fasting state in at least 25% of patients, and esophagitis was identified in 10% of patients, with no significant differences between groups (p=0.525 and p=0.098, respectively). No other lesions were detected. A repeat EGD was performed in all patients with ETI seven days after the initial examination, and in all cases, the lesions had resolved. EGD findings are summarized in Table 3.

**Table 3:** – Esophagogastroduodenoscopy results

	Group 1 (n=20)	Group 2 (n=20)	Group 3 (n=20)	p-value
ETI, n (%)	5 (25)	6 (30)	0 (0)	0.006
Grade I	0 (0)	0 (0)	0 (0)	
Grade II	1 (5)	2 (10)	0 (0)	0.250
Grade III	2 (10)	3 (15)	0 (0)	0.115
Grade IV	2 (10)	1 (5)	0 (0)	0.250
Grades III and IV, n (%)	4 (20)	4 (20)	0 (0)	0.029
Food in stomach, n (%)	5 (25)	7 (35)	8 (40)	0.525
Esophagitis, n (%)	2 (10)	2 (10)	2 (10)	0.998

ETI: esophageal thermal injury.

### Follow-up

Except for two patients in Group 2 and two in Group 3, all patients were followed for a median of 27.8 months (Q1:Q3, 24.6:31.4). A total of 13 patients (22%) experienced AF recurrence: three patients in Group 1, six in Group 2, and four in Group 3 (p=0.408). No significant differences in AF recurrence were found with respect to time to achieve PVI (p=0.648), total RF application time (p=0.480), AF classification (p=0.451), left atrial size (p=0.928), minimum RF power (p=0.658), local maximum temperature (p=0.300), or peak LET (p=0.637).

## Discussion

To the best of our knowledge, this is the first randomized trial comparing the three most common LET monitoring strategies for reducing ETI: no LET monitoring with reduced ablation power, SSP monitoring, and MSP monitoring. A low cutoff temperature (37.5 °C) was used to interrupt RF applications, and all patients underwent EGD.

### LET vs. no LET

Several studies have reported a wide range of ETI incidence during AF ablation when comparing LET monitoring to no LET monitoring. However, these studies showed mixed results, and none were randomized.^9,18-20,24-28^

Müller et al. and Kiuchi et al.^18,20^ used an MSP (SensiTherm™) thermometer and reported conflicting results: Müller’s study found a higher incidence of ETI in the temperature probe group (30% vs. 2.5%), while Kiuchi’s study reported a lower incidence (0% vs. 7.5%). More recently, Schoene et al.^29^ in the OPERA randomized controlled trial, investigated whether the use of an MSP thermometer (SensiTherm™) was noninferior to AF ablation without LET monitoring. They found the incidence of ETI was similar between the groups (11.1% vs. 8.9%). In their study, a very high temperature cutoff of 41 °C was used to stop RF applications, along with different power settings (25 to 30 W vs. 25 W), resulting in a significant difference in RF energy delivery at the posterior wall (p<0.001).

Meininghaus et al.^30^ conducted a randomized trial comparing PVI with LET monitoring using the CIRCA S-CATH™ probe versus no LET monitoring. All patients underwent EGD both before and after PVI, which was performed with a 25 W setting and a LET-guided power reduction strategy using a cutoff temperature of 41 °C. In this study, mucosal lesions were observed in six of 44 patients in the LET monitoring group and in two of 42 patients in the control group. However, the p-value was not significant.

### Type of probe

Turagam et al.^16^ assessed the thermodynamic characteristics of 22 commercially available LET probes and demonstrated a remarkably wide variation among them. The CIRCA S-CATH™ probe exhibited a superior thermodynamic profile compared to the other probes. In a prospective, nonrandomized study including 20 patients with symptomatic AF undergoing index PVI, Tschabrunn et al.^23^ showed that the CIRCA S-CATH™ had a superior thermodynamic performance compared to a standard linear, nondeflectable SSP.

In a prospective nonrandomized trial, Carroll et al.^21^ investigated the rates of ETI associated with the use of an MSP (CIRCA S-CATH™) vs. an SSP (Acoustascope, Smiths Medical ASD, Inc., Keene, NH, USA). They applied a power setting of 25 W at the posterior wall with a cutoff temperature of 38 °C. Among the 543 patients included (455 with SSP and 88 with MSP), only those with a maximum LET exceeding 39 °C underwent endoscopy (75% of patients in the MSP group and 39% in the SSP group). A significantly higher rate of esophageal ulceration was associated with the use of an MSP (46% vs. 29%, p=0.02). However, a significant difference in patient characteristics was noted, with the MSP group exhibiting a greater total RF ablation time and total energy applied.

In a randomized trial involving one hundred patients, Kuwahara et al.^22^ compared a deflectable SSP thermometer with an MSP (SensiTherm™) thermometer. An open irrigated-tip catheter without contact force sensing was used, with a RF setup of 25 to 30 W and a temperature limit of 45 °C, applying a cutoff of 42 °C. No significant difference in ETI rates was found between the groups (30% vs. 20%, p=0.25).

### Energy level

The energy level applied at the posterior wall in our study reflects the standard ablation strategy employed over the past several years.^29,31,32^ However, high-power, short-duration ablation has gained popularity in recent years. Chieng et al.^33^ published the Hi-Lo HEAT trial, which found no difference in ETI rates between 40 W and 25 W ablation strategies. Chen et al.^24^ studied 120 consecutive patients, comparing LET monitoring with the CIRCA S-CATH™ probe versus no LET monitoring, using a 39 °C cutoff to interrupt RF applications. RF power was set at 50 W throughout the procedure, with an irrigation flow rate of 20 mL/min, following a power-controlled model and guided by the ablation index (Biosense Webster), targeting a value of 400 at the left atrial posterior wall. They reported a low incidence of ETI, with no significant difference between groups (3.3% vs. 1.7%, p=0.99).

### Limitations

Our study has several limitations. It was a single-center, pilot study with a small sample size. Most patients had paroxysmal AF, a CHA_2_DS_2_-VASc score <2, and no significant left atrial enlargement. It cannot be excluded that esophageal damage may have been caused by the insertion and manipulation of the temperature probe in Groups 2 and 3, or by the transesophageal echocardiography probe. Since no EGD was performed prior to RF ablation, we can only assume that the location of the lesions in the anterior esophagus, distant from the gastroesophageal junction, makes it likely that ETI was related to AF ablation.

We did not specifically correlate local ETI findings with RF ablation data, contact force information from the electroanatomic mapping system, or temperature measurements. Only one electroanatomic mapping system was used, and different results might be observed with other systems. All ablation procedures were performed under general anesthesia. Regarding the ablation technique, neither Visitag (Biosense Webster) nor a high-power, short-duration ablation strategy was available at the time, and posterior wall isolation was performed in only one patient. We used a 30 W dragging technique, and although this approach was employed, both the recurrence rate and the incidence of esophageal lesions remained low, which might have differed had alternative strategies been used.

Regarding AF recurrence, four patients were lost to follow-up — two in Group 2 and two in Group 3 — and AF recurrence was assessed solely through electrocardiogram and 24-hour Holter monitoring.

There is no consensus regarding the optimal temperature cutoff to prevent ETI, and different cutoff temperatures were not compared in this study.

## Conclusion

This pilot study demonstrates that LET monitoring with an MSP significantly reduces the incidence of ETI compared to SSP monitoring or no monitoring, without increasing the recurrence rate of AF following RF ablation. These findings suggest that the use of an MSP thermometer can enhance patient safety during PVI by effectively minimizing the risk of ETI. However, further randomized studies with larger patient populations are needed to confirm these results and guide future clinical practice.
